# A fluid-walled microfluidic platform for human neuron microcircuits and directed axotomy†

**DOI:** 10.1039/d4lc00107a

**Published:** 2024-06-06

**Authors:** Federico Nebuloni, Quyen B. Do, Peter R. Cook, Edmond J. Walsh, Richard Wade-Martins

**Affiliations:** a Osney Thermofluids Institute, Department of Engineering Science, University of Oxford Osney Mead Oxford OX2 0ES UK edmond.walsh@eng.ox.ac.uk; b The Sir William Dunn School of Pathology, University of Oxford South Parks Road Oxford OX1 3RE UK; c Oxford Parkinson's Disease Centre and Department of Physiology, Anatomy and Genetics, University of Oxford South Park Road Oxford OX1 3QU UK richard.wade-martins@dpag.ox.ac.uk; d Kavli Institute for Neuroscience Discovery, University of Oxford, Dorothy Crowfoot Hodgkin Building South Park Road Oxford OX1 3QU UK; e Aligning Science Across Parkinson's (ASAP) Collaborative Research Network Chevy Chase MD 20815 USA

## Abstract

In our brains, different neurons make appropriate connections; however, there remain few *in vitro* models of such circuits. We use an open microfluidic approach to build and study neuronal circuits *in vitro* in ways that fit easily into existing bio-medical workflows. Dumbbell-shaped circuits are built in minutes in standard Petri dishes; the aqueous phase is confined by fluid walls – interfaces between cell-growth medium and an immiscible fluorocarbon, FC40. Conditions are established that ensure post-mitotic neurons derived from human induced pluripotent stem cells (iPSCs) plated in one chamber of a dumbbell remain where deposited. After seeding cortical neurons on one side, axons grow through the connecting conduit to ramify amongst striatal neurons on the other – an arrangement mimicking unidirectional cortico-striatal connectivity. We also develop a moderate-throughput non-contact axotomy assay. Cortical axons in conduits are severed by a media jet; then, brain-derived neurotrophic factor and striatal neurons in distal chambers promote axon regeneration. As additional conduits and chambers are easily added, this opens up the possibility of mimicking complex neuronal networks, and screening drugs for their effects on connectivity.

## Abstract 2

*In vivo*, different neurons make directional connections; however, most *in vitro* models fail to capture such connectivity. We use microfluidics with fluid walls to study neuronal circuits *in vitro* in ways that fit into existing bio-medical workflows.

## Graphical abstract

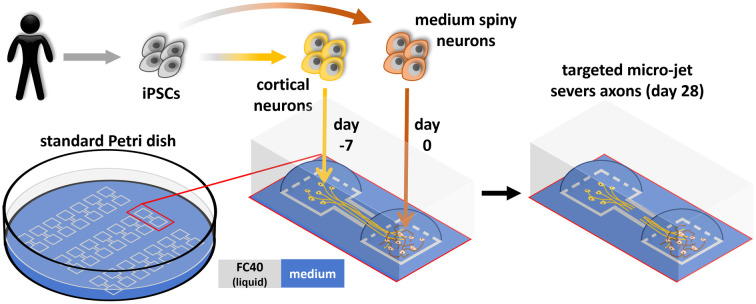

## Introduction

Various *in vitro* methods have demonstrated the requirement for complex culture systems to support neuronal maturation and the manifestation of associated disease.^1–3^ Microfluidic approaches have yielded particularly promising results, as they enable isolation of different cellular compartments (*e.g.*, somas, dendrites, axons).^4,5^ Compared to conventional *in vitro* cultures, they also permit precise control of cellular environments, and have proven useful in studies on neurotoxicity^6^ and electrical connectivity.^7,8^ Nevertheless, conventional microfluidic devices have limitations that are often attributed to the materials used for fabrication;^9^ they are usually made of a plastic elastomer (polydimethylsiloxane, PDMS) firmly bonded to a glass substrate, and cells are buried in chambers bounded by solid walls that prevent insertion of the standard experimental tools used by neurobiologists (*e.g.*, cell scrapers, patch-clamping pipettes). Consequently, neurobiologists must employ alternative protocols to use them that are different from their familiar ones.

Recently, Walsh *et al.* (2017)^23^ introduced ‘fluid-walled microfluidics’; this overcomes some of these limitations by removing most solid boundaries.^10^ It is a form of open microfluidics^10^ that exploits properties of fluids at the microscale to confine aqueous environments using interfaces (*i.e.*, fluid walls) between immiscible liquids (in this case, cell growth medium and a bio-inert fluorocarbon, FC40). Circuits can be built in minutes in standard Petri dishes, and – unlike solid walls that cannot be pierced by pipets – fluid walls allow direct access to cells everywhere in circuits. These walls re-heal automatically when pipets are withdrawn, they can be destroyed and/or reshaped without damaging cells within them,^11,12^ and are so transparent that cell morphology can be monitored using standard microscopes.^13^ These features motivate the use of this technology here.

The cortex and striatum, together with the basal ganglia and thalamus, play an important role in regulating voluntary movement, learning, executive function, and emotion.^14^ Cortical neurons (CNs) project axons toward the striatum where medium spiny neurons (MSNs) constitute up to 95% of striatal subtypes. Connectivity between CNs and MSNs is directional and monosynaptic, whilst MSNs communicate with CNs indirectly *via* downstream circuits, particularly the basal ganglia.^15^ This oriented arrangement of cortico-striatal projections is critical for their functioning but is often oversimplified by *in vitro* co-cultures.

Studies of axonal outgrowth, axotomy, and subsequent regeneration have been facilitated by the use of compartmentalised chambers that allow separation of cell bodies from their axons;^16,17^ such platforms can also allow pharmacological screening of factors acting specifically on distal axons.^16,18^ In this study, we exploit the advantages of fluid walls to establish a proof-of-concept model that recreates appropriate arrangements of human CNs and MSNs. We then go on to develop a method for targeted and localised axotomy of CNs, and compare the effects of pro-regenerative conditions on axonal regrowth;^18–20^ brain-derived neurotrophic factor (BDNF) and postsynaptic MSNs both have positive effects. In combination, these approaches provide a method for screening drugs promoting developmental outgrowth of axons and regeneration of damaged ones.

## Results

### Jet-printing of fluid-walled micro-circuits shaped like dumbbells

**Fig. 1:**
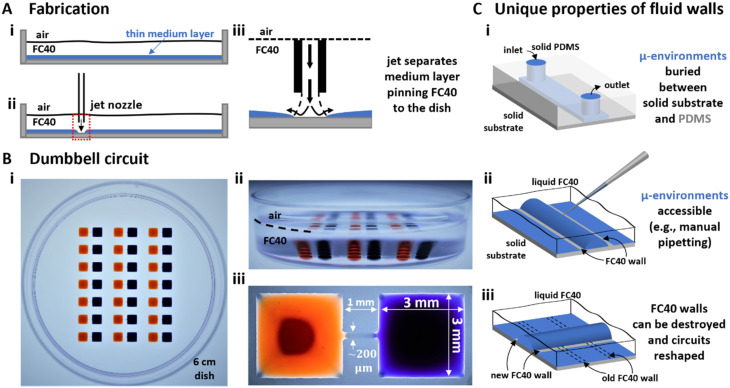
Fabrication and operation of fluid-walled circuits. (A) Fabrication. (i) In a standard polystyrene Petri dish, a thin layer of cell-culture medium is overlaid by an immiscible, transparent, and bio-inert fluorocarbon (FC40). (ii) Additional FC40 is jetted (480 μl min^−1^) through a nozzle mounted on a 3D-traverse. (iii) The submerged jet sweeps away medium, to leave fluid walls of FC40 pinned to the dish along the path of the traverse. (B) Array of dumbbells after filling each with red and blue dyes. (i and ii) Top and side views of dish. (iii) Zoomed-in image of one dumbbell (conduit length = 1 mm). (C) Comparing properties of solid PDMS walls used in conventional devices, with fluid ones. (i) Access to a conventional device is only through inlet and outlet ports. (ii) Medium can be pipetted into or out of any point in a fluid-walled circuit as liquid–liquid interfaces are easily pierced to re-heal automatically on withdrawal. (iii) Fluid walls can be destroyed at any time, and different ones recreated on demand.

The fluid-walled environment is created in standard polystyrene Petri dishes (6 cm) by ‘jet-printing’.^21^ A thin layer of cell-culture medium is overlaid by an immiscible, bio-inert, and clear fluorocarbon, FC40 (Fig. 1Ai). Next, a submerged jet of FC40 (Fig. 1Aii) sweeps medium off the plastic substrate to leave FC40 locally pinned to the dish (Fig. 1Aiii). The jetting nozzle (held by a 3-way traverse) now moves laterally above the substrate to draw the outline of the desired pattern which is held to the dish by interfacial forces acting between the two immiscible phases and the substrate. The width of the FC40 wall pinned to the dish is ∼120 μm, but it can vary depending on variables including the substrate, the velocity of the traverse, and the flow rate and size of the jet.^21^ Here, we fabricated a 7 × 3 array of dumbbell-shaped circuits in each dish; each dumbbell comprises two square chambers (3 mm) connected by a thin conduit (∼0.2 mm wide, 1 mm long, <10 μm high; Fig. 1B).

Compared to conduits in conventional devices with solid walls – where access is limited to inputs and outputs (Fig. 1Ci) – all parts of dumbbells are accessible through fluid ceilings (Fig. 1Cii). Media and/or cells are added to, and removed from, chambers by lowering a dispensing needle (also held by the traverse and connected to a syringe pump) through the FC40 until its tip are near the surface of the medium (*i.e.*, 200 μm above the bottom of the dish). Existing walls/ceilings can also be destroyed and rebuilt (Fig. 1Ciii), so circuits can be reconfigured during experiments.^11^ Additionally, fluid walls are freely permeable to vital gases, so cells in these circuits are grown in conventional CO_2_ incubators. Finally, the refractive index of FC40 (1.29) almost matches that of water (1.33), and this permits undistorted imaging with standard microscopes.^13^

### Local pressures in dumbbells

In all experiments, when cells are deposited in chambers, we require they remain there. This is impossible to achieve by rapid deposition into a newly-fabricated dumbbell, as this induces flow that carries them into the conduit^22^ (and perhaps into the other chamber). Therefore, we begin by describing how local pressures within dumbbells can be manipulated.

First consider a 1 μl drop sitting in a dish filled with FC40; the drop is shaped like the cap of a sphere with contact angle *θ* ∼ 70°,^23^ as interfacial forces minimise the contact area of medium with the immiscible fluorocarbon. Then, the pressure (*P*) at the base of the drop is defined by the Laplace pressure across the medium:FC40 interface, plus the hydrostatic head of overlying medium and FC40. The Young–Laplace equation gives Laplace pressure, 
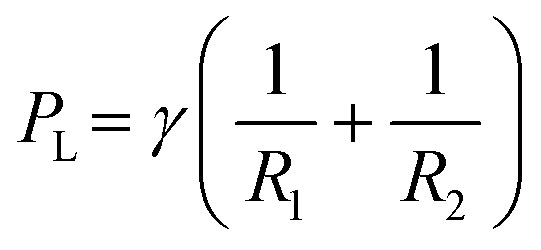
, where *γ* is the interfacial tension, and *R*_1_ and *R*_2_ are two orthogonal radii describing the curvature of the liquid wall/ceiling. Assuming our chambers have circular footprints like the drop (so 
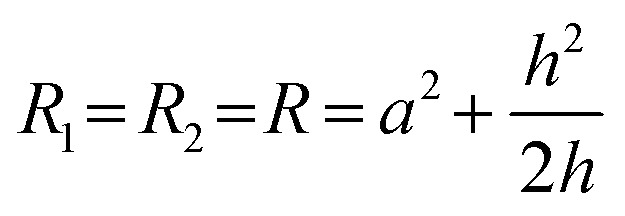
, with *a* being the radius of the chamber footprint),1
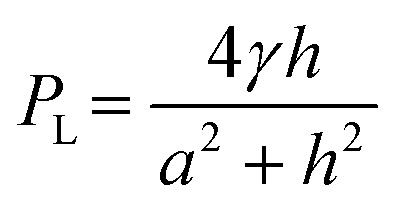
The combined pressure, *P*, at the base of a chamber then includes the two hydrostatic heads, so2
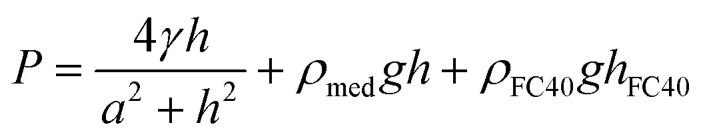
where *ρ*_med_ and *ρ*_FC40_ indicate density of medium and FC40 respectively, *g* is gravitational acceleration, *h* is drop height, and *h*_FC40_ the height of the overlay. However, when analysing the pressure difference between our two chambers (Δ*P*), the contribution of *h*_FC40_ can be expressed as the height difference of the two chambers. Therefore:3
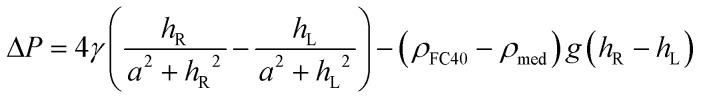
where *h*_R_ and *h*_L_ indicate right and left chamber height, respectively. *h*_R_ and *h*_L_ represent the only variables in eqn (3) when the pinning line is fixed as they depend on the volume (*V*) infused into each chamber, and4
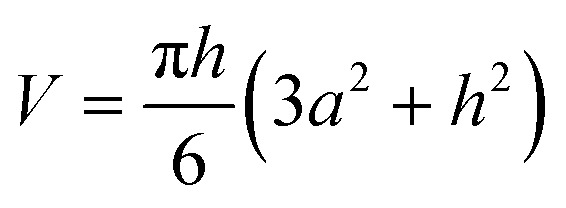
These equations are valid when chambers have the geometry of the cap of a sphere and gravity is negligible. For our case the bond number is ∼1 for chambers and Fig. S1† demonstrates the relative contribution of the Laplace and hydrostatic pressures within chambers. However, from a practical perspective we are only seeking to control the direction, rather than a defined flow rate, and this analysis shows this may be achieved simply by controlling volume addition to each chambers.

### Ensuring cells remain where deposited

**Fig. 2:**
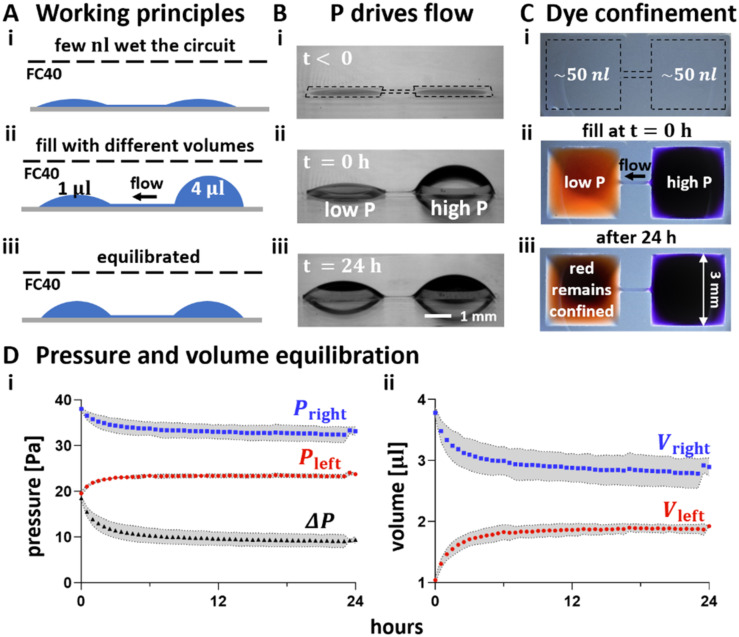
Ensuring liquid pipetted into the left of a dumbbell remains there. *P* = pressure. (A) Principles. (i) A dumbbell is flow-free after fabrication. (ii) Medium (4 μl) is added to the right-hand chamber; this generates a high local Laplace pressure. When 1 μl is immediately added to the left-hand chamber (creating a lower local Laplace pressure), resulting left-ward flow through the conduit prevents any of the 1 μl from moving to the right. (iii) Eventually the system equilibrates, and flow ceases. (B) Pressure difference drives flow. (i) Before filling. (ii) Immediately after filling with different volumes. (iii) After 24 h, chamber volumes have almost equalised due to leftward flow. (C) After adding 4 μl blue dye to the right and 1 μl red dye to the left, no red dye flows rightward (top views). (i) Before adding dyes (dotted line marks dumbbell footprint). (ii) Immediately after adding dyes. (iii) After 24 h, red dye remains confined in its chamber (which now also contains blue dye). (D) Changes in pressure (i) and volume (ii) of right- (blue) and left-hand (red) chambers determined after measuring chamber heights and calculating values using eqn (1) (*P*_right_ and *P*_left_) and eqn (3), respectively. Black curve: pressure difference between chambers. Each dot represents the mean value of 3 technical replicates, and grey areas the associated standard deviations.

After fabrication, chamber volumes are minimal, walls/ceilings are almost flat (so Laplace pressures are almost negligible), local pressures throughout a dumbbell are roughly equal, and the system is in equilibrium (Fig. 2Ai and Bi). Adding 4 μl into the right-hand chamber followed by 1 μl into the left-hand one generates a pressure difference that induces leftward flow through the conduit (Fig. 2Aii and Bii). As time passes, the system equilibrates, and volumes equalise (Fig. 2Aiii and Biii). Similarly, after adding 4 μl blue dye into a right-hand chamber and 1 μl red dye into the left-hand one (Fig. 2Ci and ii), red dye is confined to the left-hand-side for at least 24 h (Fig. 2Ciii; note the conduit is filled with blue dye, and the left-hand chamber contains both dyes). Quantification of chamber pressures and volumes over time confirm that both converge towards equilibrium values, and that blue pressure is always greater than red pressure over 24 h (Fig. 2D). This confirms that if the right-hand chamber is pre-filled with 4 μl before adding 1 μl to the left-hand one, there cannot ever be flow rightward. The cell-substrate adhesion is routinely observed to occur within an hour of cell plating, with increasing strength over time.^24^ We exploited these phenomena to ensure that cells are seeded in the selected chamber, they remain there.

When both chambers have equal volumes, their internal pressures are also equal – and so there is no flow in either direction. As a result, mass transport between chambers only occurs by diffusion to generate a concentration gradient in the conduit, with the steepness and duration of the gradient depending on dumbbell geometry and diffusion constant (see ESI†). Fig. S2 and Table S1† show how such a gradient of BDNF changes over time.

### Axons outgrow from CNs through the conduit to the distal chamber

Axon pathfinding is led by a variety of molecular cues, both intrinsic^25^ and target-derived.^26^ While the roles of intrinsic factors have been investigated using dissociated cultures of neurons *in vitro*,^20,27^ less is known about target-derived signals due to difficulties in recreating the required micro-environments around neurons. Here, we developed a microfluidic model that recreates such an environment; we exploited the intrinsic physics of fluid-walled dumbbells (Fig. 2), and established a workflow (Fig. S3†) that confines CNs in the left-hand chamber as axons grow through the conduit to the right-hand (distal) one.

**Fig. 3:**
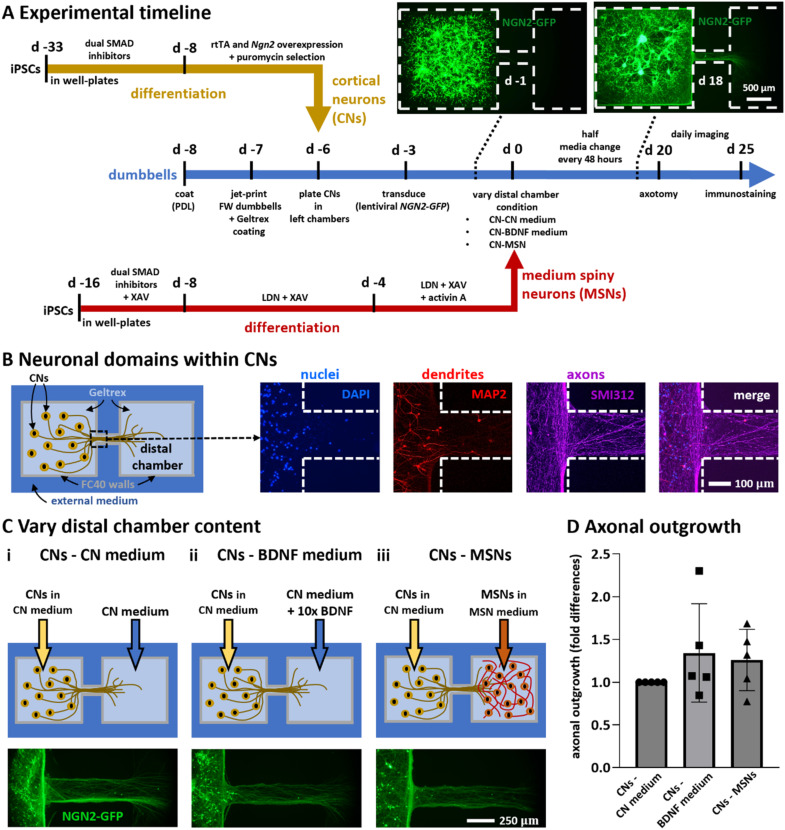
Timelines and culture conditions used to model and study unidirectional outgrowth of cortical neurons. (A) Timelines. Yellow and red lines show protocols for generating CNs and striatal MSNs from iPSCs using standard methods; blue line describes those involved in making and operating fluid-walled dumbbells. Insets: After plating transduced CNs in left-hand chambers, live-cell images (collected on d −1 and 18) show NGN2-GFP fluorescence in parts of dumbbells (dotted white lines show circuit edges). (B) Immunostaining shows compartmentalisation of neuronal domains in CNs (d 25). Cartoon: CNs in left-hand chamber, CN medium (but no MSNs) in distal one. Nuclei (DAPI) and MAP2-positive dendrites are confined within the chamber, while only SMI312-positive axons grow into the conduit (dotted white lines show circuit edges). (C) Effects of varying distal-chamber content on axonal outgrowth. In all cases, CNs expressing NGN2-GFP are plated in CN medium in left-hand chambers. Top: Cartoons indicating chamber contents. Bottom: Live-cell epifluorescence images of left end of conduit (d 20); axons extend into conduits in all three cases. (i) Monoculture control. (ii) Positive control with regenerative medium in distal chamber (CN medium + 10-fold higher concentration of 100 ng ml^−1^ BDNF). (iii) MSNs + MSN medium in distal chamber (this condition attempts to promote connectivity between cortex and striatum seen *in vivo*). (D) Quantitative analysis of axonal outgrowth seen in (C). Outgrowth (fold difference) is difference in area covered by GFP-expressing neurites in conduits between d 0 and d 20, normalised for GFP-positive conduit area on d 0 as a function of the number of cells, and expressed relative to their control. Each dot represents a healthy control-derived line from one differentiation. *N* = 2 iPSC lines, *n* = 2–3 differentiations per line. One-way ANOVA with Bonferroni correction; *p* > 0.05.

Fig. 3A provides an overview of the 3 major workflows that are now used. In one (yellow arrow), human induced pluripotent stem cells (iPSCs) were induced to differentiate into (post-mitotic) CNs using proven methods in standard well plates.^28^ In a second (red arrow), human iPSCs were similarly induced to develop into post-mitotic MSNs, again using conventional methods.^29^ In a third (blue arrow), fluid-walled dumbbells were jet-printed in 6 cm polystyrene Petri dishes, and then CNs and MSNs are plated into dumbbells.

In all experiments that will be described, CNs are deposited into left-hand chambers of dumbbells (using conditions established in Fig. 2), and transduced 3 days later with lentiviruses encoding (tetracycline-inducible) neurogenin-2-GFP (NGN2-GFP) to allow live-cell visualisation of transduced CNs. During subsequent culture (with CN medium in the right-hand chamber but no cells), axons (now expressing NGN2-GFP) grow through the conduit into the distal chamber (Fig. 3A, insets; compare live-cell images on days −1 and 18). In some cases, different media and/or MSNs were deposited on d 0 in the right-hand (distal) chamber. To ensure that deposited MSNs remained where deposited, we again exploited the approach described in Fig. 2, except that now it is the left-hand chamber that was pre-filled with 4 μl before 1 μl of cell suspension is plated in the distal one.

As we wish to replicate the spatial organisation of CNs and MSNs *in vivo*, we require that CN somas remain confined to the left-hand chamber, and that only axons grow into the conduit. We confirmed successful compartmentalisation by immunostaining (d 25) using domain-specific markers: DAPI-labelled nuclei and MAP2-positive dendrites were confined to the left-hand chamber, while the conduit was populated with SMI312-positive axons (Fig. 3B).

We have seen that after plating in the left-hand chamber, post-mitotic CNs – with standard CN (maturation) medium in both chambers – started projecting axons into the conduit between d −6 to d 0. We then varied contents of the distal chamber at d 0 to see what effects concentration gradients of diffusing molecules along the conduit have on axonal outgrowth. This was tested using three different conditions. First, a control where CNs project axons towards distal CN medium – so there is no chemical gradient in the conduit (CNs-CN medium condition). A second condition where the distal chamber was filled with CN medium supplemented with 10x concentration (100 ng ml^−1^) of BDNF (CNs-BDNF medium condition). Third, a more physiological condition where CNs extended their projections towards a population of MSNs (CNs-MSNs condition).

Axonal outgrowth in each of the 3 conditions was quantified as the difference of areas covered by GFP-expressing neurites in conduits measured at d 0 and d 20 (Δ*A* = *A*_d20_ − *A*_d0_). As Δ*A* may vary depending on the number of cells in each chamber making comparison between dumbbells difficult, we divided it by *A*_d0_ to normalise results decoupling them from the effective count of CNs seeded. Additionally, to compare results from different batches of differentiated iPSCs, axonal outgrowths in the CNs-BDNF medium and CNs-MSNs conditions were expressed as the fold-difference of the CNs-CN medium control of the respective batch. Perhaps surprisingly, neither condition significantly increased axonal outgrowth (Fig. 3D; *p* > 0.05, one-way ANOVA with Bonferroni correction) suggesting it occurred independently of exogenously-supplied BDNF or MSN-derived molecules.

### A unidirectional circuit between cortex and striatum

**Fig. 4:**
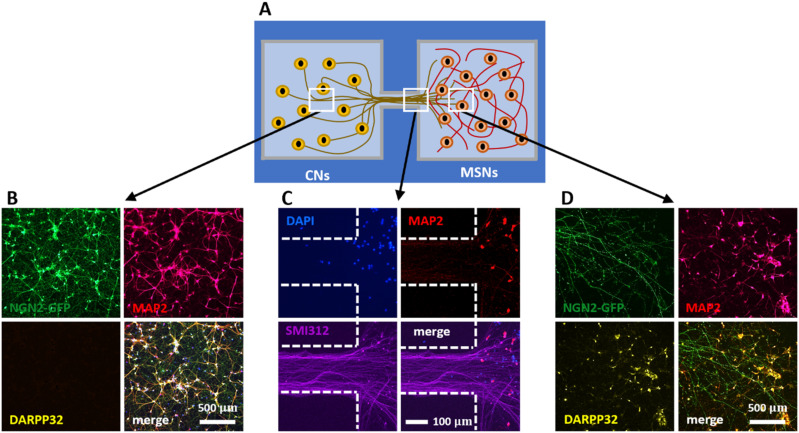
Modelling unidirectional pathway between cortex and striatum. Cortical axons marked with NGN2-GFP grow from the left chamber through the conduit into the distal chamber containing MSNs (DAPI – blue – nuclei, MAP2 – red – dendrites, SMI312 – purple – axons, NGN2-GFP – green – transduced CNs, DARPP32 – yellow – MSN marker; merges indicated). (A) Top-view schematic of the cortex-striatum circuit in a fluid-walled dumbbell. CNs are seeded in the left chamber and cultured for 6 days, then MSNs are plated in the distal (right) chamber; after growth for another 25 days, cells are immunolabelled. White boxes indicate areas imaged below using various markers. (B) MAP2-positive dendrites and NGN2-GFP are found throughout the CN chamber (but not DARPP32-expressing MSNs). (C) MSN nuclei remain where seeded and develop MAP2-positive dendrites; they are joined by SMI312-positive CN axons from the left-hand chamber. (D) Axons containing NGN2-GFP from transduced CNs are intertwined amongst MAP2- and DARPP32-positive neurons in the striatal chamber.

The CNs-MSNs condition described above provides an *in vitro* model for possible connectivity between cortex and striatum. This prompted a more detailed immunolabelling analysis on d 25 (Fig. 4). Note here that NGN2-GFP (now detected using an anti-GFP antibody) is a CN-specific marker, and DARPP32 is a MSN-specific one; MAP2 and SMI312 mark respectively dendrites and axons in both cell types. As expected, CNs in the left-hand chamber expressed NGN2-GFP plus MAP2, but no (striatal-specific) DARPP32 (Fig. 4A and B). The right-hand end of the conduit contained no nuclei stained with DAPI (confirming somas remain where plated), but many SMI312-positive axons that appeared to invade the distal chamber with its MAP2-positive dendrites (Fig. 4C). The distal chamber was populated by many (CN-derived) NGN2-GFP-positive axons that ramified amongst MSNs expressing (striatal-specific) DARPP32 plus MAP2. These results are consistent with no spill-over of somas from either chamber, and unidirectional growth of CN axons into the distal chamber where they ramify amongst MSNs.

### Axotomy using a micro-jet

**Fig. 5:**
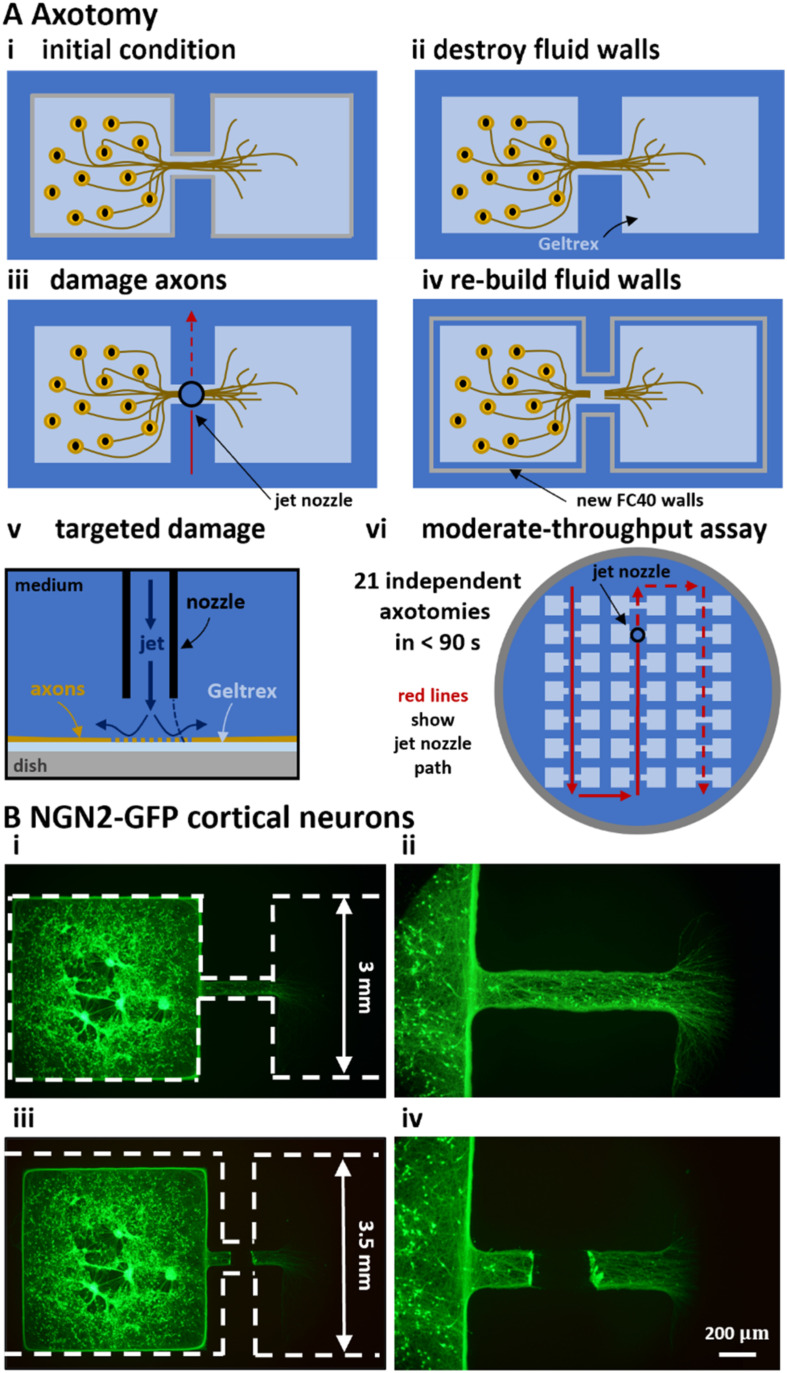
Axotomy assay on fluid-walled microfluidics. (A) Schematics illustrating localized axotomy. (i) Circuit before starting axotomy. (ii) Fluid walls are destroyed; cells remain attached to Geltrex within the dumbbell footprint. (iii) The traverse moves a nozzle that jets medium onto axons and cuts them. (iv) New fluid walls are jet-printed (using an FC40 jet) to form a new dumbbell slightly larger than the original one; after filling the new dumbbell, axons regrow. (v) Higher-magnification schematic of the submerged media jet in action targeting axons to generate localised damage. (vi) Overview. Red lines illustrate the path followed by the medium jet nozzle while damaging 21 dumbbells (7 × 3 array) in a single 6 cm Petri dish in less than 90 seconds. Solid lines indicate the path already covered; dashed lines show future nozzle positions. (B) Representative live-cell fluorescent images of CNs expressing NGN2-GFP pre- and post-axotomy. (i and ii) Dashed lines mark edges of dumbbell footprints before and after axotomy. (iii and iv) Higher magnification images taken before and after axotomy.

We next used a micro-jet to sever axons projecting from CNs through a conduit into an empty distal chamber (Fig. 5Ai). The process involved three steps. First, fluid walls were destroyed by removing (manually by pipet) the FC40 overlay, and adding ∼5 ml medium to the dish; CNs and axons remained attached to the substrate (Fig. 5Aii). Second, a submerged jet of medium (emitted from the same nozzle used for FC40 jet-printing) was moved perpendicularly by the traverse across the axons to sever them (Fig. 5Aiii). Third, new fluid walls were built just outside the original ones to recreate a slightly-larger dumbbell (Fig. 5Aiv). To do so, most medium was gently removed to leave a thin layer, fresh FC40 was overlaid, and a new dumbbell jet-printed around the original one so that the FC40 stream did not impinge on existing attached cells or axons (Fig. S3C†). This technique allows targeted axotomy of a chosen segment within an axon (Fig. 5Av). Moderate-throughput axotomy in each of the 21 original dumbbells in one dish is achieved in <90 s (Fig. 5Avi). Comparison of live-cell images of cortical axons expressing NGN2-GFP taken before and after axotomy revealed how local the damage was, with the jet clearing the axotomized area of most cellular material (Fig. 5Bi and ii).

### Regeneration after axotomy

**Fig. 6:**
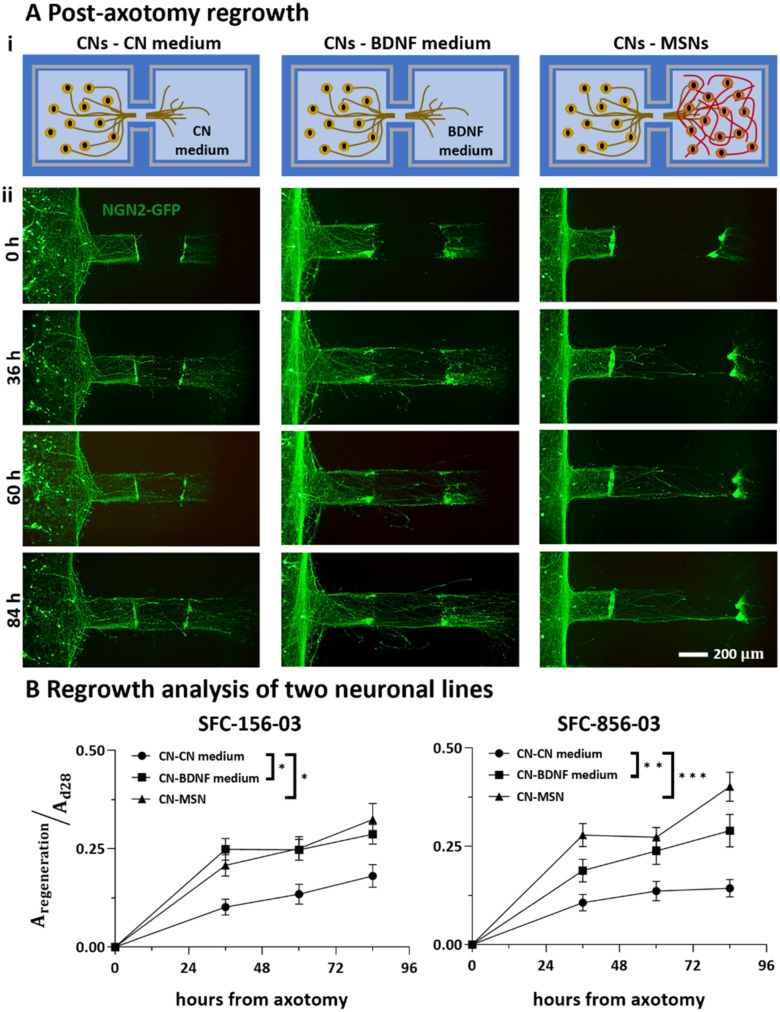
Effects of distant target-derived factors on regrowth of cortical axons after damage. (A) Schematic and representative live-cell fluorescent images of CNs expressing NGN2-GFP taken 0–84 h after axotomy. (B) Fraction of axotomized area re-covered by axons derived from two different healthy control iPSC lines (SFC-156-003, SFC-856-03). *N* = 2–3 differentiations per iPSC line, * *p* < 0.05, ** *p* < 0.01, *** *p* < 0.001, two-way ANOVA with Bonferroni correction test. (SFC156-03: CN-CN medium *versus* CN-BDNF medium, *p* > 0.05 at 36 and 60 hours, *p* < 0.01 at 84 hours; CN-CN medium *versus* CN-MSN, *p* < 0.0001 at 36 hours, *p* < 0.001 at 60 and 84 hours; SFC856-03: CN-CN medium *versus* CN-BDNF medium, *p* < 0.001 at 36 hours, *p* < 0.05 at 60 and 84 hours; CN-CN medium *versus* CN-MSN, *p* < 0.01 at 36 and 84 hours, *p* < 0.05 at 60 hours; two-way ANOVA with Bonferroni correction test). Furthermore, BDNF and MSNs exert comparable positive effect on cortical axonal regeneration (for both iPSC lines, CN-BDNF medium *versus* CN-MSN *p* > 0.05, two-way ANOVA with Bonferroni correction test).

After printing new dumbbells around damaged cultures, the three conditions were re-established with equal volumes of correct media in each respective chamber (Fig. 6Ai), and then regeneration of axons was monitored for 5 more days (till d 25). As only the original footprint of the dumbbells was coated with Geltrex, axonal regrowth was directed over the same areas rather than the larger footprint of the new dumbbells. Representative immunostaining on d 25 shows the axotomized area of the conduit refilled with axons only expressing NGN2-GFP and SMI312, but not the dendrite-specific MAP2 (Fig. S4†). As regrowth after axonal damage may involve target-derived signalling,^30,31^ we compared effects of our three conditions on this regeneration (Fig. 6Ai).

As for outgrowth, regeneration was quantified as the area covered by GFP-expressing neurites growing into a rectangular zone within the cleared area (Fig. 6Aii; axon tracts that are incompletely severed or not fully cleared of cells are excluded from this analysis). This zone abuts the proximal damage line and has the same width of the original conduit (*i.e.*, 200 μm) while extending 300 μm into the cleared area. Results were normalised against values seen prior to axotomy on d 20 to account for differences in axonal numbers and transduction efficiency. Results obtained with our two different iPSC lines from healthy donors (again with 2–3 differentiations per line) show that both BDNF and MSNs enhance regeneration to roughly the same degree compared to the control (Fig. 6B). These results show that severed CN axons can regenerate, and that BDNF and MSNs enhance this.

## Discussion

Our strategic goal is to develop microfluidic methods facilitating the production of, and experimentation on, neuronal circuits of any type *in vitro*; critically, we require that these methods fit easily into bio-medical workflows. We described proof-of-principle experiments illustrating individual steps towards this end. We began by fabricating in minutes dumbbell-shaped micro-circuits in standard Petri dishes (6 cm; Fig. 1), and developed conditions ensuring that cells plated in one or other chamber remain where deposited (Fig. 2). Next, post-mitotic CNs derived from human iPSCs were seeded in left-hand chambers of the dumbbell, so axons could grow through connecting conduits of 1 mm to distal chambers (Fig. 3A and B). Outgrowth of cortical axons was compared against three different targets in the distal chamber (Fig. 3C and D); results showed such outgrowth was not enhanced by BDNF. This result aligns with a previous study on mouse primary dorsal route ganglia (DRG) cultures^32^ in which high levels of BDNF did not boost normal distal growth. Another study^33^ observed that bathing mouse primary cortical neurons in 50 ng ml^−1^ BDNF increased axonal outgrowth by ∼25%. In our study, BDNF was applied distally and the CNs bodies were exposed to a maximum of 15 ng ml^−1^ BDNF, allowing for diffusion of BDNF from the distal chamber (see ESI†), likely explaining the difference in results. Although the BDNF signalling pathway is likely conserved across species,^31^ as far as we are aware, this is the first report on the influence of external diffusible factors applied distally on axonal outgrowth of human CNs *in vitro*; hence, modulation by BDNF as well as other neurotrophins will require further investigation. Interestingly, as MSNs also showed no effect on normal healthy outgrowth, we hypothesise axonal outgrowth of post-mitotic human iPSC-derived CNs to be independent of endogenous postsynaptic targets.

We then developed a circuit in which axons project from CNs in the left-hand chamber, through the conduit, and on to ramify amongst MSNs in the distal one (Fig. 4). We anticipate such circuits will prove especially useful for studying cortical-striatal connectivity because they are so accessible to biologists. For example, an obvious next step is to examine electrical connectivity using patch clamping^29^ and/or super-resolution of pro calcium imaging;^34^ our circuits can be built on glass surfaces and incorporated into both workflows without modification.

Similar to previous work,^32,35^ we also developed a moderate-throughput axotomy assay in which axons growing through conduits are severed by a fluid jet (Fig. 5). In contrast to existing methods that use mechanical stresses,^36,37^ vacuum aspiration^18,38,39^ or toxins^40^ for axotomy – all procedures that might be expected to yield poor reproducibility^41^ – we hope our (arguably non-contact) method will yield more consistent results. In our assay the quality of the axotomy depends on cells density and age as these parameters affect the thickness of the axonal bundle; ‘cut’ parameters like jet flow rate, nozzle height above the dish, and traverse speed were finely tuned to achieve the desired result. We noticed axotomy performed on the CNs-MSNs micro-circuit consistently resulted in a wider ‘cut’ with the right end of the damage almost always corresponding to the beginning of the distal chamber where cortical axons meet the medium spiny neurons. This phenomenon requires further investigation, but we hypothesized it to be caused by the asymmetry of the adhesion mechanism on the two sides of the axotomy (left: cortical axons on Geltrex, right: axons intertwined with striatal processes). Using this assay, we showed that BDNF or MSNs in distal chambers promote axon regrowth (Fig. 6).

Here, the heightened demand for BDNF following axon injury^42^ might explain the observed positive effect on axonal regeneration despite the neutral effect shown on axonal outgrowth. Co-culture of CNs with their striatal target MSNs also promoted cortical axonal regeneration post-axotomy comparable to that induced by exogenous BDNF, suggesting that the distant postsynaptic target could release pro-regenerative factors acting to facilitate reinnervation.

In conclusion, we have recreated a basic micro-circuit containing human neurons that mimics the unidirectional connectivity seen between cortex and striatum *in vivo*, as well as developing a moderate-throughput axotomy assay. Our approaches benefit from the intrinsic advantages provided by fluid walls that include ease of circuit fabrication and operation, plus compatibility with existing bio-medical workflows. As additional conduits and chambers can be easily added, we anticipate these approaches will expand the experimental toolkit available for the study of human neuronal networks in health and disease.

## Materials and methods

### Fluorocarbon 40 (FC40)

FC40 was purchased from 3 M. FC40^STAR^ (iotaSciences Ltd, Oxfordshire, UK) is a compound treated with a proprietary method to improve formation of fluid walls. Throughout the article, the term ‘FC40’ is used to refer to FC40^STAR^. FC40 has a vapour pressure of 432 Pa at 25 °C (ref. 10) and hence low evaporation rate, 2 ml in a 6 cm dishes lasting for several days. The solubility of water in FC40 is ∼7 ppm (ref. 23) and so effectively prevents loss of media by diffusion from filled chambers. Although FC40 is totally bio-inert and so bio-compatible, it has a high global warming potential and recycling/disposal should be done in accordance with manufacturers recommendations.

### The fluid printer

The printer (iotaSciences Ltd, Oxfordshire, UK) consists of a 3D traverse with two printing heads (two blunt needles of different internal diameters) plus built-in software. Each needle is connected to a syringe pump through Teflon tubes. One needle (70 μm internal diameter) is attached to a syringe containing FC40 and it is used for jet-printing; this is the jetting needle. The other (dispensing) needle (255 μm internal diameter) is attached to a syringe normally filled with ethanol to guarantee sterility; it handles cells and media samples. Both needles can be moved above the surface of a Petri dish as pumps infuse liquids.

In our design, 21 dumbbells were printed in each dish by the jetting needle on d −7. Thereafter, every volume infusion/removal from chamber was operated by the dispensing needle. Every operation on one chamber of dumbbells (either left or right), was repeated to all dumbbells in the dish before starting any task on the opposite chamber.

### Generation of iPSC-derived post-mitotic cortical neurons

This is the yellow pathway in Fig. 3. Generation of CNs from two iPSC control lines from healthy human patients SFC156-03-01 (EBiSC409 cat# STBCi101-A, RRID:CVCL_RD71), and SFC856-03-04 (RRID:CVCL_RC81) was adapted from an established protocol.^28^ In brief, neuronal development was induced with dual SMAD inhibitors 10 μM SB431542 (Tocris) and 118 nM LDN (Sigma) for 25 days (d −33 to d −8). These progenitors (d −8) were transduced with lentiviruses that encoded a doxycycline-inducible tetO promoter driving constitutive expression of rtTA and mouse neurogenin-2 (Ngn2).^43^ A puromycin resistance gene was also co-expressed to select for cells expressing Ngn2.

### Generation of iPSC-derived post-mitotic medium spiny neurons

This is the red pathway in Fig. 3. Two iPSC control lines (SFC156-03-01 and SFC856-03-04) were differentiated into MSNs using conditions modified from established protocols.^29^ In brief, neural induction of sub-pallial identity was initiated by dual SMAD inhibitors – 10 μM SB431542 (Tocris) and 118 nM LDN (Sigma) – and inhibition of WNT signalling – 4 μM XAV (Tocris) – from d −16 to d −8. From d −8 to d 0, SB431542 was removed and neurogenesis in the culture mediated by LDN and XAV. Activin A (25 μg ml^−1^; SKU# SRP3003), the key regulator of TGF-β signalling, was added from d −4 to d 0.

### Fabrication of dumbbells

This is the blue pathway in Fig. 3 from d −8 to d −6. On d −8, 6 cm Petri dishes were pre-coated with 7 ml of poly-d-lysine (0.01 mg ml^−1^) overnight. On d −7, dishes were washed twice with PBS and subsequently loaded with Neurobasal medium (1 ml; ThermoFisher) supplemented with 1× B27 (ThermoFisher). After at least 5 minutes, medium was manually removed to leave a thin layer (∼50 μl) attached to the bottom of the dish, and FC40 (∼2 ml) gently pipetted on to this thin layer. An array of 7 × 3 dumbbells was jet-printed using a fluid printer (iotaSciences Ltd.).^21^ When used with cells, dumbbell chambers were each loaded with 2 μl Geltrex™ (0.46 mg ml^−1^; ThermoFisher) and incubated at 37 °C overnight (to d −6). Geltrex was infused into dumbbells simply to confine the coating within the patterned footprint. This promoted post-axotomy regrowth only over the same area rather than that of the slightly larger dumbbell created damaging axons. Chambers with right-angled footprints provide several advantages over ones with curved footprints, including increased speed of manufacture, and higher density of micro-environments per dish as they can easily tessellate.

### Maturation media used for culturing in dumbbells

Cortical maturation medium (CN medium) used from d −6 to d 25 and beyond: Neurobasal, 1× B27 with vitamin A, 1× Glutamax, penicillin/streptomycin (50 U or mg per ml) (1 : 200), 1 mg ml^−1^ doxycycline (1 : 1000), 10 ng ml^−1^ BDNF, 10 ng ml^−1^ NT-3 (1 : 1000), 200 ng ml^−1^ laminin (1 : 5000), 200 mM ascorbic acid (1 : 1000). This corresponds to Ngn2 base medium from protocol https://doi.org/10.17504/protocols.io.bp2l69qr5lqe/v1.

Cortical maturation medium + 10× BDNF (BDNF medium) used from d 0 to d 25 and beyond: CN medium with 100 ng ml^−1^ BDNF.

Striatal maturation medium (MSN medium) used from d 0 to d 8: DMEM/F12 basal medium, 1% MEM non-essential amino acids (NEAA), 1% l-glutamine, 1× B27 without vitamin A, 1% penicillin/streptomycin (P/S), 0.05% β-mercaptoethanol. This corresponds to striatal maturation medium 1 from protocol https://doi.org/10.17504/protocols.io.eq2ly79prlx9/v1.

Striatal maturation medium (MSN medium) used from d 8 to d 25: 50% DMEM/F12 basal medium, 50% Neurobasal, 1% MEM non-essential amino acids (NEAA), 1% l-glutamine, 1× B27 plus vitamin A, 1% penicillin/streptomycin (P/S), 0.05% β-mercaptoethanol. This corresponds to striatal maturation medium 2 from protocol https://doi.org/10.17504/protocols.io.eq2ly79prlx9/v1.

### Plating CNs in left-hand chambers and transduction

This is the blue pathway from d −6 to d 0. On day −6, 2 μl was removed from each chamber, 4 μl cortical-maturation media deposited in the right-hand chamber (to create a positive pressure gradient toward the left chamber), and post-mitotic CNs plated (1 μl containing 13 000 cells in cortical maturation medium) in each left-hand chamber. On day −3, CNs were transduced by adding 2 μl lentivirus encoded *NGN2-GFP* in cortical maturation medium (50 μl of viral stock/2 μl) to the left-hand chamber, and 1 μl fresh medium without lentiviruses to the right-hand one (to give ∼4 μl per chamber). On d −2, 4 μl were removed from each chamber and 4 μl fresh medium added back (to wash away free lentivirus).

### Varying conditions on d 0

On d 0, both chambers initially contained ∼4 μl cortical maturation medium, with CNs in the left-hand one. Then, contents of the distal chamber were varied to give the 3 conditions (CNs-CN medium, CNs-BDNF medium, and CNs-MSNs). For all conditions, 4 μl was removed from both chambers, and 4 μl CN medium added to the left-hand chamber. Next, 1 μl of either CN medium, or CN medium plus 100 ng ml^−1^ BDNF, or a MSNs suspension (13 000 cells in MSN medium) was added to the distal chamber. As before, a pressure difference confined MSNs and BDNF to right-hand chambers.

### Culturing CNs in dumbbells without MSNs after d 0

This is the blue pathway from d 0 to d 25 and beyond for the CNs-CN medium and CNs-BDNF medium conditions. On d 2, both chambers were completely emptied (removing ∼4 μl from each chamber). Then, 4 μl CN medium was added to the CN chamber and 4 μl of either CN or BDNF medium to the distal one (to give 4 μl per chamber). Every 48 h thereafter, half the specified medium present in a chamber was replenished by removing 2 μl spent medium and then adding back 2 μl fresh medium of the same kind.

### Culturing CNs in dumbbells with MSNs after d 0

This is the blue pathway from d 0 to d 25 and beyond for the CNs-MSNs condition. On d 2, both chambers were completely emptied (removing ∼4 μl from each chamber). Then, 4 μl of fresh CN medium was added to CN chamber and 4 μl of MSN medium containing 200 nM cytosine arabinoside (araC) to arrest proliferation of any non-neuronal cells in the population to the distal one (to give 4 μl per chamber). Every 48 h thereafter, half the medium in each chamber was changed by withdrawing 2 μl and adding an equal volume of fresh medium; this gradually diluted araC over MSNs. On d 8, a full medium change is done to switch MSNs to a new MSN medium (see maturation media used for culturing in dumbbells).

### Axotomy

Fluid walls were destroyed by gently pouring all FC40 out of the dish followed by two washes with cortical maturation medium (care is taken to prevent cell/axon peeling). Directed axotomy was performed automatically using the fluid printer by modifying an existing procedure.^44^ A 1 ml glass syringe (Hamilton) filled with Neurobasal medium (ThermoFisher) was connected *via* Teflon tubes to the jetting nozzle. Then, a medium jet was ejected (480 μl min^−1^) from the nozzle (70 μm inner diameter) held 0.3 mm above the dish as the traverse moved (960 mm min^−1^) the nozzle in a straight line perpendicular to the axons' main direction of outgrowth (Fig. 4B). Following axotomy, dishes were re-filled with fresh FC40, and new fluid walls jet-printed around the original footprint. The new dumbbells had larger footprints (chamber area = 3.5 × 3.5 mm^2^, conduit length = 0.5 mm, width ∼400 μm) to avoid damaging attached cells/axons. 4 μl fresh cell medium was deposited into each chamber.

Fig. 6 summarises data obtained from 2–3 differentiations per cell line, and measurements from 100–150 dumbbells/differentiation. Another ∼30% dumbbells were discarded due to incomplete severing or clearing of axons in the axotomised area of the conduit, and/or incomplete rebuilding of new dumbbells (which results in media leakage) [fluid walls are almost always built successfully on virgin Petri dishes, but success rates are lower when building on dishes that have been covered with a thin skim of medium overlaid with FC40 from d −7 to d 20].

### Imaging

All fluorescent live-cell images of dumbbells were taken with a digital single-lens reflex camera (Nikon D7100 DSLR) connected to an epifluorescence microscope (Olympus IX53). Images were analysed using Cell Profiler 3.8 (RRID:SCR_007358) to describe and quantify axon outgrowth and regrowth.

### Immunostaining

All FC40 was discarded from the dish, cultures washed twice with PBS, fixed (2% paraformaldehyde, 20 min at room temperature, RT), and washed three times with PBS. Fixed samples were then incubated (80 °C, 5 min) in citrate buffer pH 6.0 (ThermoFisher), and left for 10 min at RT. Permeabilization and blocking were performed concurrently in PBS, 10% donkey serum, and 0.01% Triton X-100 for 10 min. Following incubation with primary antibody in PBS and 10% donkey serum overnight at 4 °C, samples were washed with PBS, and incubated in species-appropriate Alexa Fluor© secondary antibody in PBS with 10% donkey serum for 1 hour at RT. Tables S2 and S3† list antibodies used. Images are acquired on an Invitrogen EVOS™ FL Auto (ThermoFisher) cell-imaging system, and processed using ImageJ (RRID:SCR_003070).^45^

### Estimating pressures and volumes in chambers

Dumbbells were printed in wide rectangular plates (Thermo Scientific™ Nunc™ Rectangular Dishes single well) to improve imaging from the side, as the curved plastic walls of 6 cm dishes distort views. Chambers were jet-printed as before after filling a dish with ∼5 ml DMEM + 10% FBS, removing all but a thin film, and overlaying ∼10 ml FC40. Jet printing used a fluid printer modified to host rectangular dishes (Hylewicz CNC-Technik). The same medium was infused into chambers by a syringe pump (PhD Ultra, Harvard Apparatus) equipped with a 50 μl glass syringe (Hamilton) connected to a blunt metal needle (33G blunt NanoFil™ needle, World Precision Instruments) through a Teflon tube (Zeus Company Inc.) to generate the desired initial volume/pressure difference. Volumes were dispensed following the same sequence used during experiments with neurons. Thus, 4 μl were initially infused in the right-hand chamber, followed by 1 μl into the left-hand one. Images of the two chambers equilibrating were recorded from the side every 30 min for 24 h using a camera (FTA1000 B Class, First Ten Angstrom) placed perpendicular to the connecting conduit (Fig. 2B). Chambers heights are measured using FTA32 software (First Ten Angstrom, RRID:SCR_024392) and the outer diameter of the dispensing needle (210 μm) as a scale reference. Heights were converted into pressures and volumes (Fig. 2D) using eqn (1) and (2), respectively.

### Statistical analysis

All data were presented as mean ± standard error of the mean (SEM) unless otherwise stated. Raw data were tested for normality and statistical comparison of the means is performed using one- or two-way ANOVA with Bonferroni *post hoc* test; a difference is considered significant if *p* < 0.05. All statistical analyses were performed on GraphPad Prism 6.0 (GraphPad Software, RRID:SCR_002798).

## Conclusion

We provided a mechanism using fluid-walls to fabricate *in vitro* microenvironments to model the directional connectivity between cortex and striatum using standard Petri-dishes. We first established conditions to confine cortical neurons to the left-hand side of a dumbbell (using Laplace pressure to control local pressures). Then, axons grow to the right through the connecting conduit into the other chamber. When medium spiny (striatal) neurons are deposited on the right-hand side, immunolabelling shows that axons derived from cortical neurons grow through the conduit to ramify amongst the striatal ones. Additionally, we developed a localised axotomy assay: connecting axons are severed by a microjet of medium. Subsequently, axons regrow to the right, and this regrowth is affected by the presence of different neurotrophic factors deposited in the right-hand chamber. In combination, these techniques should permit the development of neuronal circuits of increasing complexity useful for the analysis of neural growth and connectivity, as well as for the recovery after damage.

## Author contributions

F. N. and Q. B. D. conceived the project. F. N. and Q. B. D. designed, performed, and analysed all experimental data. P. R. C., E. J. W., and R. W. M. supervised the study. F. N. and Q. B. D. prepared the first draft of the manuscript. All authors reviewed the manuscript and approved its submission.

## Conflicts of interest

P. R. C. and E. J. W. co-founded, and hold equity in, IotaSciences Ltd. The same company provides financial support to F. N., plus reagents and equipment for the study. Oxford University Innovation – the technology transfer company of The University of Oxford – has filed patent applications on behalf of P. R. C. and E. J. W. covering technologies used in this study.

## Supplementary Material

LC-024-D4LC00107A-s001

## Data Availability

The data that support the findings of this study are deposited on Zenodo (DOI https://doi.org/10.5281/zenodo.7924430). All details of the antibodies, cell lines, virus strains, and software used in this work are available in a key resources table deposited in Zenodo (DOI https://doi.org/10.5281/zenodo.7924430). Protocols associated with this work can be found on protocols.io (DOI https://doi.org/10.17504/protocols.io.36wgqjwwxvk5/v2), and additional details are available in a key resources table deposited in Zenodo (DOI https://doi.org/10.5281/zenodo.7924430). All original code used to jet-print the microfluid-walled dumbbells is available at GitHub (https://github.com/craggASAP/microfluid_axotomy.git) and has been deposited in Zenodo (DOI https://doi.org/10.5281/zenodo.11148154).
